# Endoscopic features associated with hospitalization outcomes in IgA vasculitis patients: a single-center retrospective cohort study

**DOI:** 10.3389/fimmu.2026.1731886

**Published:** 2026-04-20

**Authors:** Quxin Juhou, Yunfang Song, Ruiheng Xie, Dongqing Wang, Jinxi Zhao, Xiaomin Shi, Wei Zhang, Zhongqiong Wang, Xiaohong Wang, Xiaowei Tang

**Affiliations:** 1 Department of Gastroenterology, the Affiliated Hospital of Southwest Medical University, Luzhou, China; 2 Department of Gastroenterology, Xuzhou Central Hospital, Xuzhou Clinical School of Xuzhou Medical University, Xuzhou, China; 3 Department of Endoscopic Center, the Affiliated Hospital of Southwest Medical University, Luzhou, China

**Keywords:** association, endoscopy, gastrointestinal involvement, IgA vasculitis, length of stay, multi-segment involvement, ulceration

## Abstract

**Objectives:**

To evaluate associations between endoscopic features and hospitalization outcomes in IgA vasculitis (IgAV) with gastrointestinal (GI) involvement, focusing on endoscopic subtypes associated with prolonged hospitalization.

**Methods:**

We analyzed 132 IgAV patients with GI involvement and complete endoscopic data at a large-volume center (January 2019 to December 2024). Clinical manifestations, endoscopic characteristics, laboratory data, and abdominal imaging findings at admission were reviewed. Prolonged length of stay (LOS) was defined as LOS exceeding the 75th percentile (>13 days). Multivariable logistic regression and gamma regression (log link) were used to examine factors associated with prolonged LOS and LOS as a continuous outcome. Sensitivity analyses additionally adjusted for treatment variables and renal involvement. Laboratory parameters were compared across endoscopic subgroups in exploratory analyses.

**Results:**

The median age of the patients was 18 years (IQR: 15.0–45.5), with a male-to-female ratio of 2:1. Abdominal pain was the most common presenting symptom. Bowel wall thickening was the most frequent finding on abdominal imaging. The most prevalent endoscopic finding was congestion/edema, followed by erosions, ulcers, and petechiae/ecchymosis. Endoscopic ulceration (OR 2.40, 95% CI 1.09–5.27, p=0.029) and multi-segment GI involvement (OR 2.58, 95% CI 1.20–5.58, p=0.016) were independently associated with prolonged LOS. Gamma regression showed that ulceration was associated with a 27.7% longer LOS (RR 1.277, 95% CI 1.047–1.564, p=0.018) and multi-segment involvement with a 34.7% longer LOS (RR 1.347, 95% CI 1.035–1.748, p=0.025). The association with ulceration remained significant after adjusting for treatment factors, whereas the association with multi-segment involvement was attenuated. The association of ulceration with LOS was more pronounced in patients aged ≤18 years. In sensitivity analyses, additional adjustment for renal involvement did not materially change the associations between endoscopic features and LOS.

**Conclusions:**

In IgAV with GI involvement, endoscopic ulceration is the endoscopic feature most consistently associated with longer hospitalization, and the association appears stronger in patients aged ≤18 years. Multi-segment involvement shows a weaker association that attenuates after treatment adjustment. Overall, these findings support the clinical value of endoscopic phenotyping to inform clinical assessment and should be interpreted as associations rather than prognostic predictions.

## Introduction

Immunoglobulin A vasculitis (IgAV), formerly known as Henoch-Schönlein purpura, is an acute, systemic, immune complex-mediated vasculitis of small vessels characterized by IgA-dominant immune complex deposition. There are four main presenting symptoms and signs: palpable purpura without thrombocytopenia or coagulopathy; arthritis and arthralgia; abdominal pain, sometimes associated with bleeding; and renal disease (IgA nephropathy) that presents with proteinuria or hematuria (1–6). Although IgA vasculitis is typically self-limited and IgAV spontaneously resolves in 94 percent of children and 89 percent of adults (3–6), gastrointestinal and renal complications may pose a severe threat to the lives of patients with IgAV.

While more commonly diagnosed in children, adult-onset IgAV is increasingly recognized as a distinct entity with a more severe clinical course, particularly regarding gastrointestinal (GI) and renal involvement (7–9). GI symptoms occur in up to 50–70% of adults with IgAV, often manifesting as acute abdominal pain, bleeding, nausea, or diarrhea. In approximately 50% of cases, these symptoms may even precede the classic cutaneous purpura, posing significant diagnostic challenges and potentially delaying appropriate treatment (10–12).

The diagnosis of IgAV remains primarily clinical, supported by histopathological evidence when available. In recent years, abdominal computed tomography (CT) and endoscopy have emerged as valuable tools for evaluating GI involvement. CT - and endoscopy-based intestinal evaluation can aid in the diagnosis of the disease, especially for patients presenting with predominantly abdominal symptoms and without typical purpuric skin rash. Studies have found that endoscopic features including diffuse mucosal redness, petechiae, erosion, and ulceration, with the duodenum and small intestine being the most commonly affected segments (8–10). CT imaging provided objective insights into the extent of intestinal involvement, and patients with widespread bowel involvement displayed a more severe disease state (13).

Furthermore, laboratory parameters, such as elevated white blood cells (WBC) count, D-dimer (D-D), C-reactive protein (CRP) and neutrophil-to-lymphocyte ratio (NLR) have been associated with GI involvement (14, 15). Despite these advances, significant gaps remain in current understanding. While prior studies have described imaging and endoscopic features in IgAV patients, few have established a clear link between these findings and clinically meaningful outcomes such as disease severity, hospitalization duration, or resource utilization. The prognostic value of endoscopic phenotypes—such as ulcerative lesions or multi-segment involvement—remains poorly defined, limiting their utility for clinical risk assessment and management planning.

Therefore, this study aimed to bridge this gap by conducting a detailed analysis of endoscopic characteristics in a well-defined cohort of patients with IgAV and GI involvement. We sought to determine whether specific endoscopic features were associated with disease severity and prolonged hospitalization. Additionally, we integrated laboratory and imaging data to examine their associations with hospitalization outcomes in patients with abdominal IgAV. We hypothesized that endoscopic evaluation may provide complementary information on disease behavior beyond diagnostic assessment, potentially informing clinical monitoring and decision-making.

## Methods

### Study design and population

This single-center retrospective cohort study was conducted at a high-volume tertiary hospital from January 2019 to December 2024. We enrolled 132 consecutive patients diagnosed with IgAV and confirmed GI involvement, all of whom underwent comprehensive endoscopic evaluation during their hospitalization. The diagnosis of IgAV was established according to the criteria set forth by the American College of Rheumatology (ACR) or the European Alliance of Associations for Rheumatology (EULAR), in collaboration with the Pediatric Rheumatology International Trials Organization (PRINTO) and the Pediatric Rheumatology European Society (PRES) (16–18). Intestinal involvement was defined by abdominal pain, nausea or vomiting, diarrhea, bowel obstruction, intussusception, gastrointestinal bleeding, intestinal ulcers, intestinal perforation, and intestinal necrosis. Exclusion criteria encompassed malignancies, viral hepatitis, hematologic disorders, and incomplete endoscopic data. The study protocol received ethical approval from the Institutional Review Board (No. KY2025303), with waived informed consent due to the retrospective anonymized design.

### Data collection and variables

Data on demographic characteristics, clinical symptoms, laboratory parameters, abdominal imaging findings, and endoscopic features were systematically collected from electronic medical records. Clinically relevant variables included age, sex, symptom profile—encompassing purpura, abdominal pain, hematochezia, and arthralgia—as well as the duration of hospitalization. Laboratory measures consisted of WBC count, NLR, platelet count (PLT), CRP, D-dimer, fibrinogen, albumin, and immunoglobulin levels (IgA, IgG, IgM, IgE). Imaging features were assessed via abdominal CT or MRI, with specific attention to bowel wall thickening, mesenteric abnormalities, and lymph node enlargement. Endoscopic records were evaluated for the presence of mucosal abnormalities—such as congestion, edema, petechiae, erosions, and ulcers—as well as signs of bleeding, the number of affected gastrointestinal segments (categorized as 1, 2, or ≥3), and the segmental involvement of specific regions including the stomach, duodenum, jejunum/ileum, colon, and rectum. A single independent researcher, blinded to clinical outcomes, conducted this retrospective assessment using standardized reports and images. Validation followed a rigorous three-step protocol: initial alignment with original descriptors, subsequent contextual clarification through clinical notes for ambiguous cases, and systematic documentation of all classification decisions. Data on treatments administered during the hospitalization were systematically extracted. This included the use of systemic corticosteroids, other immunosuppressants, and nutritional support. These variables were collected to account for potential confounding in the association between endoscopic findings and hospitalization duration. In addition, baseline renal indicators (GFR, serum creatinine, and urinalysis for proteinuria and hematuria) were collected to address potential confounding by renal involvement. Urinalysis results were recorded using routine semi-quantitative grading, and renal involvement was defined as proteinuria ≥1+ and/or hematuria ≥1+ and/or abnormal kidney function, operationalized as reduced GFR or elevated serum creatinine based on institutional reference ranges. Renal involvement was included as an additional covariate in sensitivity analyses.

### Primary outcomes and statistical analysis

The primary outcome was prolonged hospitalization, defined as a length of stay (LOS) exceeding the 75th percentile for the cohort (>13 days). Gamma regression (log link) was used to model LOS as a continuous outcome. Continuous variables are summarized as median with interquartile range (IQR) or mean ± standard deviation (SD), depending on their distribution as assessed by the Shapiro-Wilk test. Categorical variables are expressed as frequencies and percentages. Group comparisons involved the use of independent t-tests or Mann-Whitney U tests for continuous variables, and Chi-square or Fisher’s exact tests for categorical variables. Univariate logistic regression was performed to identify candidate variables associated with prolonged LOS, with results expressed as odds ratios (ORs) and 95% confidence intervals (CIs). Sensitivity analyses were conducted by additionally adjusting the gamma regression models for treatment variables (systemic corticosteroids, other immunosuppressants, and nutritional support) and renal involvement. Additionally, multivariable gamma regression with a log-link function was used to estimate the percent change in LOS associated with various endoscopic features, adjusted for age and sex. Laboratory comparisons across endoscopic subgroups (ulceration status, segment extent, and bleeding) were conducted as exploratory analyses using tests appropriate to distribution; given missingness and the exploratory nature, results were interpreted descriptively and were not adjusted for multiple comparisons. A two-tailed p-value < 0.05 was considered statistically significant. All analyses were conducted using R software (version 4.4.3).

## Results

### Patient characteristics and features of imaging

**Figure 1:**
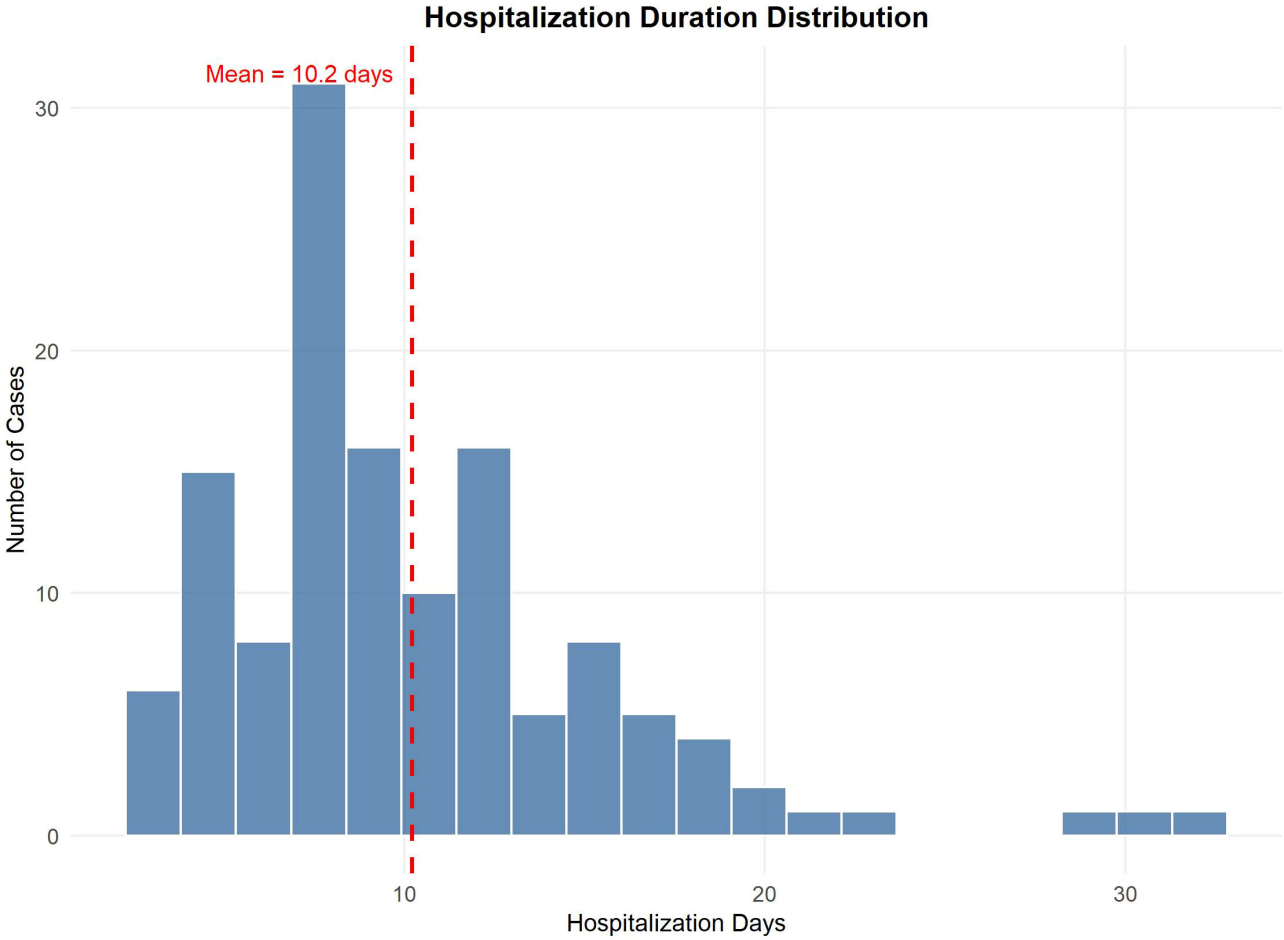
Hospitalization duration distribution. The histogram displays the frequency of patients (Y-axis) relative to their length of hospital stay in days (X-axis). The distribution is right-skewed, indicating that a majority of patients experienced comparatively shorter hospitalizations. The red dashed vertical line denotes the mean hospitalization duration of 10.2 days. This visualization underscores the variability in length of stay, a key metric for assessing hospitalization burden and healthcare resource utilization within this patient population.

A total of 132 patients were enrolled, and their demographic characteristics and clinical manifestations are shown in **Table 1**. Patients presented with a median age of 18.0 years (interquartile range [IQR] 15.0–45.5), and among the enrolled patients, 88 patients (66.7%) were male and 44 patients(33.3%) were female, with a male-to-female ratio of 2:1. Abdominal pain was the most common clinical manifestation (116 [87.9%]), followed by cutaneous purpura (98 [74.2%]), hematochezia (41 [31.1%]), and nausea or vomiting (44 [33.3%]). Diarrhea was reported in 19 patients (14.4%). Arthralgia/arthritis was observed in 16 patients (12.1%). Among the 83 patients who underwent abdominal imaging, bowel wall thickening was the most frequent finding (40 [48.2%]), followed by lymph node abnormalities (17 [20.5%]) and mesenteric alterations (15 [18.0%]). The distribution of hospitalization duration for the entire cohort is shown in **Figure 1**. The LOS distribution was right-skewed, indicating that most patients experienced relatively short hospital stays, with a mean duration of 10.2 days.

**Table 1:** Baseline characteristics and imaging findings. (n = 132).

Items	N (%) or median (IQR)
Age, years	18.0 (15.0-45.5)
Sex	
Male	88 (66.7%)
Female	44 (33.3%)
Symptom	
Purpura	98 (74.2%)
Abdominal pain	116 (87.9%)
Hematochezia	41 (31.1%)
Nausea or vomiting	44 (33.3%)
Diarrhea	19 (14.4%)
Arthralgia/arthritis	16 (12.1%)
Imaging manifestations	83
Bowel-wall thickening	40 (48.2%)
Lymph node changes	17 (20.5%)
Mesenteric changes	15 (18%)

### Endoscopic findings

Endoscopic evaluations, detailed in **Table 2**, revealed near-universal mucosal congestion and edema (131 [99.2%]). Erosions were documented in 91 patients (68.9%) and ulcerations in 35 (26.5%). The stomach was the most commonly involved segment (123 [93.2%]), followed by the duodenum (88 [66.7%]). Involvement of the jejunum and ileum (31 [23.5%]), rectum (21 [15.9%]), and colon (4 [3.0%]) was also observed. Multi-segment involvement was frequent, occurring in 106 patients (80.3%); specifically, 68 (51.5%) had two affected segments and 38 (28.8%) had three or more segments involved. Single-segment involvement was noted in 25 patients (18.9%). Active bleeding was documented in 37 patients (28.0%).

**Table 2:** Features of endoscopic findings.

Endoscopy manifestations	N (%)
Mucosal lesion types	
Congestion and edema	131 (99.2%)
Petechia/ecchymosis	6 (4.5%)
Erosion	91 (68.9%)
Ulceration	35 (26.5%)
Number of involved segments	
1 segment	25 (18.9%)
2 segments	68 (51.5%)
≥3 segments	38 (28.8%)
Lesion distribution	
Stomach	123(93.2%)
Duodenum	88(66.7%)
Jejunum/Ileum	31(23.5%)
Colon	4(3.0%)
Rectum	21(15.9%)
Bleeding signs	
No bleeding	95 (72.0%)
Bleeding	37 (28.0%)

### Laboratory parameters

Key laboratory findings are presented in **Table 3**. Because several laboratory variables were available only for subsets of patients, results are summarized according to the “Available, n/N” denominator. The median WBC was 12.41 (IQR, 9.20–17.32) × 10^9/L (available, 131/132), and 89/131 (67.9%) had WBC >10 × 10^9/L. The median NLR was 7.52 (IQR, 4.27–12.27) (available, 131/132), and 112/131 (85.5%) had NLR >3. The median platelet count was 307.00 (IQR, 248.00–372.50) × 10^9/L (available, 131/132), with 25/131 (19.1%) having PLT >400 × 10^9/L. CRP was available for 12/132 patients, with a median of 25.65 (IQR, 10.60–57.85) mg/L; 10/12 (83.3%) had CRP >5 mg/L. D-dimer was available for 51/132 patients, with a median of 5.17 (IQR, 1.97–9.43) μg/mL; 49/51 (96.1%) had D-dimer >0.5 μg/mL. Albumin was available for 132/132 patients, with a median of 40.95 (IQR, 36.98–45.40) g/L; 22/132 (16.7%) had albumin <35 g/L. Median creatinine was 60.50 (IQR, 46.60–68.30) μmol/L (available, 131/132). Complement and immunoglobulin profiles were available only in subsets of patients (C3/C4: 26/132; IgA/IgG/IgM: 27/132). Urinalysis identified hematuria in 50/102 (49.0%) and proteinuria in 59/102 (57.8%).

**Table 3:** Laboratory findings.

Parameter	Value, median (IQR)	Available, n/N
WBC (×10^9/L)	12.41 (9.20-17.32)	131/132
WBC >10	89 (67.9%)	
NLR	7.52 (4.27-12.27)	131/132
NLR >3	112 (85.5%)	
PLT (×10^9/L)	307.00 (248.00-372.50)	131/132
PLT>400	25 (19.1%)	
PDW (fL)	15.90 (15.65-16.20)	131/132
MPV (fL)	9.40 (8.55-10.40)	131/132
CRP (mg/L)	25.65 (10.60-57.85)	12/132
CRP >5	10 (83.3%)	
D-dimer (μg/mL)	5.17 (1.97-9.43)	51/132
D-dimer >0.5	49 (96.1%)	
ALB (g/L)	40.95 (36.98-45.40)	132/132
ALB <35	22 (16.7%)	
Crea (μmol/L)	60.50 (46.60-68.30)	131/132
Urea (mmol/L)	4.67 (3.70-6.03)	131/132
IgA (g/L)	2.70 (2.31-3.04)	27/132
IgG (g/L)	9.64 (8.02-10.40)	27/132
IgM (g/L)	0.81 (0.54-1.22)	27/132
IgE (IU/mL)	89.20 (69.45-280.25)	12/132
C3 (g/L)	1.14 (0.96-1.29)	26/132
C4 (g/L)	0.26 (0.19-0.34)	26/132

Data are presented as median (interquartile range). The “Available, n/N” column indicates the number of patients with available data out of the total cohort (N = 132). Abnormal values (presented as n (%)) are defined based on institutional reference ranges. ALB, albumin; CRP, C-reactive protein; MPV, mean platelet volume; NLR, neutrophil-to-lymphocyte ratio; PDW, platelet distribution width; PLT, platelet; WBC, white blood cell.

### Endoscopic features associated with prolonged hospitalization

**Figure 2:**
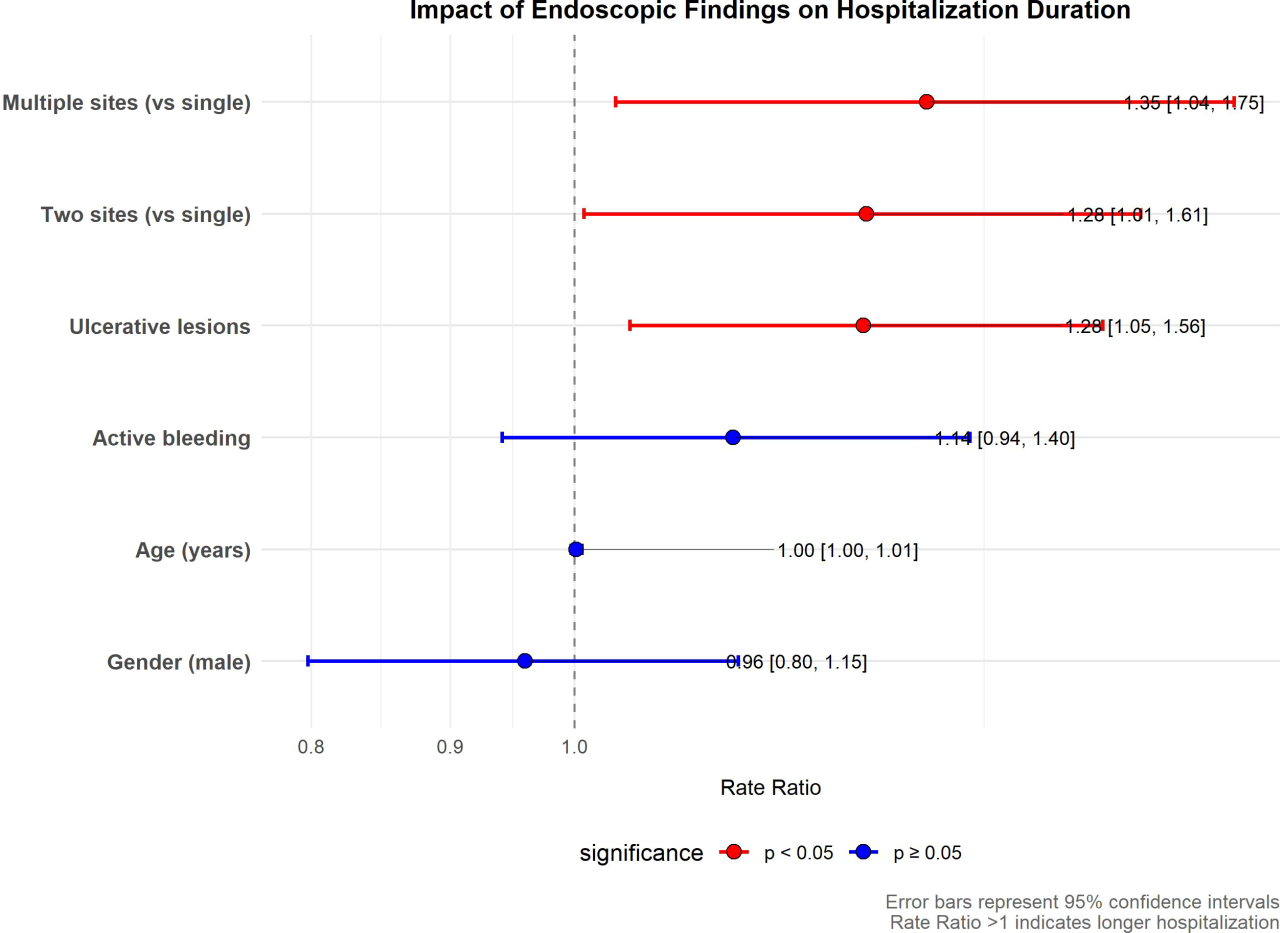
Impact of endoscopic findings on hospitalization duration. The plot displays rate ratios (RR) with 95% confidence intervals (CIs) for the association between selected covariates—including endoscopic features and demographic factors—and hospitalization length. The vertical line at RR = 1 represents the line of no effect. Each estimate is plotted as a point (RR) with a horizontal line (95% CI). Statistically significant associations (p < 0.05) are highlighted in red, and non-significant associations (p ≥ 0.05) are shown in blue. Key findings: Ulcerative lesions (RR: 1.28, 95% CI: 1.05–1.56), two-segment involvement (RR: 1.28, 95% CI: 1.01–1.61), and multi-segment involvement (RR: 1.35, 95% CI: 1.04–1.75) were significantly associated with longer hospitalization. In contrast, active bleeding (RR: 1.14; 95% CI: 0.94–1.40), age (RR: 1.00; 95% CI: 1.00–1.01), and male gender (RR: 0.96; 95% CI: 0.80–1.15) showed no statistically significant association. These results highlight the association between endoscopic findings and hospitalization duration.

Univariate logistic regression analyses identified several endoscopic features significantly associated with hospitalization duration. The presence of ulcerative lesions (odds ratio [OR] 2.40; 95% confidence interval [CI] 1.09–5.27; p = 0.029) and multi-segment involvement (≥3 segments) (OR 2.58; 95% CI 1.20–5.58; p = 0.016) were identified as factors associated with prolonged hospitalization, whereas single-segment involvement was associated with lower odds (OR 0.29; 95% CI 0.10–0.83; p = 0.021) (**Table 4**). To quantify these associations with LOS as a continuous outcome, gamma regression with a log-link function was performed (**Table 5**). The analysis confirmed that ulcerative lesions were associated with a 27.7% longer LOS (rate ratio [RR] 1.277; 95% CI 1.047–1.564; p = 0.018). Similarly, involvement of three or more segments was associated with a 34.7% longer LOS (RR 1.347; 95% CI 1.035–1.748; p = 0.025), while two-segment involvement was associated with a 28.0% longer LOS (RR 1.280; 95% CI 1.008–1.615; p = 0.041). The forest plot in **Figure 2** provides a consolidated visual representation of these effect estimates, clearly illustrating the point estimates and confidence intervals for all covariates and affirming the statistical significance of the key endoscopic predictors. In contrast, active bleeding (RR 1.144; 95% CI 0.940–1.397; p = 0.170), age (RR 1.001; p = 0.584), and male gender (RR 0.959; p = 0.648) demonstrated no statistically significant association with hospitalization length.

**Table 4:** Univariate analysis of endoscopic features and prolonged hospitalization.

Item	OR (95%CI)	P
Mucosal lesion types		
Congestion and edema	0.46 (0.19-1.14)	0.093
Petechia/ecchymosis	2.19 (0.35-13.58)	0.399
Erosion	0.81 (0.41-1.63)	0.563
Ulceration	2.40 (1.09-5.27)	**0.029**
Number of involved segments		
1 segment	0.29 (0.10-0.83)	**0.021**
2 segments	0.99 (0.49-1.96)	0.966
≥3 segments	2.58 (1.20-5.58)	**0.016**
Bleeding signs		
No bleeding	1.45 (0.65-3.26)	0.365
Bleeding	3.80 (0.71-20.35)	0.119

Bold values indicate statistical significance (p < 0.05).

**Table 5:** Gamma regression analysis for hospital stay duration.

Gamma regression results (log link)					
Predictor	Rate ratio	95% CI lower	95% CI upper	% change	P-value
Intercept	7.251	5.710	9.304	Reference	<0.001
Ulcerative lesions	1.277	1.047	1.564	27.7%	0.018
Two segments (vs single)	1.280	1.008	1.615	28.0%	0.041
Multiple segments (vs single)	1.347	1.035	1.748	34.7%	0.025
Active bleeding	1.144	0.940	1.397	14.4%	0.170
Age (years)	1.001	0.997	1.006	0.1%	0.584
Gender (male)	0.959	0.798	1.148	-4.1%	0.648

Gamma regression model with log link function. Rate Ratio (RR) > 1 indicates an increase in length of stay (LOS), while RR < 1 indicates a decrease. The “% Change in LOS” is calculated as (RR - 1) × 100%, representing the percentage increase or decrease associated with the predictor relative to the reference (Intercept).

### Sensitivity and exploratory analyses

To address potential confounding by therapeutic interventions and cohort age heterogeneity, complementary analyses were performed. After adjusting for treatment variables (systemic corticosteroids, other immunosuppressants, and nutritional support) in multivariable gamma regression models, endoscopic ulceration remained significantly associated with prolonged length of stay (LOS) (adjusted RR 1.214; 95% CI 1.005–1.473; p = 0.046), while multi-segment involvement (≥3 segments) showed attenuated significance and did not reach conventional statistical significance (adjusted RR 1.236; 95% CI 0.965–1.579; p = 0.087) (**Table 6**).

**Table 6:** Sensitivity analysis: gamma regression adjusted for treatment factors.

Variable	Rate ratio (95% CI)	% change	P-value
Intercept	4.759 (3.354 to 6.878)	375.9%	<0.001
Ulcerative lesions	1.214 (1.005 to 1.473)	21.4%	0.046
Two segments (vs single)	1.176 (0.940 to 1.464)	17.6%	0.150
Multiple segments (vs single)	1.236 (0.965 to 1.579)	23.6%	0.087
Active bleeding	1.153 (0.960 to 1.391)	15.3%	0.121
Age (years)	1.002 (0.998 to 1.007)	0.2%	0.331
Gender (male)	0.972 (0.819 to 1.149)	-2.8%	0.740
Systemic corticosteroids	1.642 (1.286 to 2.073)	64.2%	<0.001
Other immunosuppressants	1.256 (0.802 to 2.090)	25.6%	0.347
Nutritional support	1.026 (0.748 to 1.377)	2.6%	0.866

Model adjusted for systemic corticosteroids use, other immunosuppressants use, and nutritional support. Gamma regression model with log link function, adjusted for treatment factors. The model was adjusted for the use of systemic corticosteroids, other immunosuppressants, and nutritional support. Rate Ratio (RR) > 1 indicates an increase in length of stay (LOS).

*Post-hoc* stratification using the median age of 18 years revealed differential effects across age groups: In the pediatric/adolescent subgroup (≤18 years), the ulcer-LOS association persisted (RR 1.309; 95% CI 1.015–1.703; p = 0.043). Conversely, adults (>18 years) demonstrated a directionally consistent association (RR 1.165; 95% CI 0.881–1.547) that did not reach statistical significance (p=0.284) (**Table 7**). In additional sensitivity analyses adjusting for renal involvement, the estimated associations between endoscopic features and LOS were not materially changed (**Table 8**), and renal involvement was not independently associated with LOS in that model.

**Table 7:** Age-stratified analysis: association between endoscopic ulceration and hospitalization duration.

Variable	Group A RR (95% CI)	Group A P-value	Group B RR (95% CI)	Group B P-value
Ulcerative lesions	1.309 (1.015 to 1.703)	0.043	1.165 (0.881 to 1.547)	0.284
Two segments (vs single)	1.226 (0.912 to 1.632)	0.169	1.089 (0.774 to 1.517)	0.619
Multiple segments (vs single)	1.231 (0.891 to 1.690)	0.205	1.256 (0.854 to 1.840)	0.233
Active bleeding	0.870 (0.678 to 1.128)	0.286	1.459 (1.132 to 1.889)	0.007
Gender (male)	0.868 (0.683 to 1.097)	0.244	1.004 (0.773 to 1.296)	0.976
Systemic corticosteroids	1.603 (1.108 to 2.259)	0.009	1.728 (1.235 to 2.375)	0.002
Other immunosuppressants	1.607 (0.877 to 3.305)	0.158	0.864 (0.435 to 1.905)	0.688
Nutritional support	1.049 (0.630 to 1.653)	0.840	0.916 (0.600 to 1.360)	0.662

Group A: ≤ 18 years (n = 72), Group B: > 18 years (n = 60). Models adjusted for systemic corticosteroids use, other immunosuppressants use, and nutritional support. Gamma regression models with log link function, stratified by age (≤18 vs. >18 years). All models were adjusted for the use of systemic corticosteroids, other immunosuppressants, and nutritional support. Rate Ratio (RR) > 1 indicates an increase in length of stay (LOS).

**Table 8:** Renal sensitivity model (with renal involvement).

Variable	Rate ratio (95% CI)	% change	P-value
Ulcerative lesions	1.275 (1.044 to 1.558)	27.5%	0.019
Two sites (vs single)	1.269 (1.007 to 1.600)	26.9%	0.046
Multiple sites (vs single)	1.333 (1.033 to 1.720)	33.3%	0.029
Active bleeding	1.142 (0.943 to 1.385)	14.2%	0.177
Age (years)	1.001 (0.996 to 1.006)	0.1%	0.634
Gender (male)	0.962 (0.804 to 1.152)	-3.8%	0.677
Renal involvement	1.012 (0.833 to 1.229)	1.2%	0.904

Renal sensitivity model additionally adjusted for renal involvement derived from baseline GFR/creatinine and urinalysis (“-” negative; “—” missing).

### Laboratory correlates of endoscopic subtypes

In exploratory subgroup comparisons (**Supplementary Tables S1–S3**), laboratory markers are reported with their corresponding available sample size because immune and inflammatory tests were obtained in only subsets of patients. Patients with ulcerative lesions exhibited significantly higher platelet counts (345.17 ± 92.09 vs. 306.07 ± 86.24 × 10^9^/L; p = 0.026) and fibrinogen levels (4.79 ± 1.07 vs. 3.92 ± 1.16 g/L; p < 0.001) compared to those without ulcers. No statistically significant differences were observed in available immunoglobulins (IgA/IgG/IgM) or complement levels (C3/C4) by ulceration status; however, these comparisons were limited by small sample sizes (**Supplementary Table S1**). Multisegment involvement (≥3 segments) was associated with neutrophilia (13.27 ± 7.02 vs. 10.63 ± 5.67 × 10^9^/L; p = 0.035), hypoalbuminemia (38.58 ± 5.41 vs. 41.62 ± 5.83 g/L; p = 0.009), reduced IgG levels (7.44 ± 2.48 vs. 9.75 ± 2.19 g/L; p = 0.036), and hyponatremia (136.87 ± 2.91 vs. 138.55 ± 3.71 mmol/L; p = 0.014) relative to limited (≤2 segments) involvement. Patients with active bleeding demonstrated elevated alanine aminotransferase (26.68 ± 24.39 vs. 16.00 ± 11.05 U/L; p = 0.003), increased gamma-glutamyl transferase (42.64 ± 46.97 vs. 27.46 ± 27.80 U/L; p = 0.006), and lower albumin levels (38.90 ± 6.08 vs. 41.36 ± 5.70 g/L; p = 0.039) compared to those without bleeding.

## Discussion

Although IgAV is typically self-limiting, some patients may experience life-threatening visceral involvement. Therefore, early recognition and treatment are crucial for improving the prognosis of patients with IgAV (16–19). However, there have been patients with abdominal manifestations in whom the typical skin rash was lacking at onset, posing a considerable diagnostic challenge for clinicians (8, 20–22). Therefore, familiarity with characteristic endoscopic findings and careful observation of all GI findings may be particularly helpful when gastrointestinal symptoms precede cutaneous findings.

This study provides a comprehensive analysis of endoscopic characteristics in a large cohort of patients with IgAV involving the gastrointestinal tract. By employing duration of hospitalization as a clinically relevant composite endpoint reflecting disease severity, treatment response, and complication development, we identified specific endoscopic findings—namely, the presence of ulcerations and involvement of three or more gastrointestinal segments—as independent associates of prolonged hospital stay. Importantly, because LOS is also influenced by treatment intensity and institutional practice, these findings should be interpreted as associations rather than direct measures of biologic severity. These results support the potential clinical utility of endoscopy beyond diagnostic confirmation, suggesting that certain endoscopic phenotypes may help identify patients more likely to require longer hospitalization.

The topographic distribution of gastrointestinal lesions observed in our cohort is consistent with a high frequency of stomach (93.2%) and duodenum (66.7%) involvement, reflecting IgAV’s recognized predilection for the upper GI tract (20, 23). Upper gastrointestinal involvement was frequent in this cohort (87% endoscopic abnormalities), consistent with findings from a large French multicenter study of adult IgA vasculitis (n=260) (24). The study underscores the clinical significance of gastrointestinal involvement, noting that while clinical responses and recurrence rates were similar between patients with and without gastrointestinal manifestations, those with such involvement typically received higher doses of treatment. These findings align with current perspectives on the role of endoscopy in IgA vasculitis management, highlighting its transition from diagnostic evaluation to early risk assessment. This underscores the importance of endoscopic assessment in understanding the clinical spectrum and prognostic factors associated with gastrointestinal involvement in IgA vasculitis (25). The nearly universal presence of congestion/edema (99.2%) and the high prevalence of erosions (68.9%) further reflect the diffuse inflammatory nature of this condition. More importantly, our identification of ulceration and multi-segment involvement as endoscopic phenotypes associated with prolonged hospitalization provides clinicians with objective, quantifiable descriptors that may help contextualize the expected inpatient course.

Gastrointestinal involvement in IgAV patients is associated with elevated levels of WBC, D-dimer, CRP, Neutrophils and Lymphocytes (14, 26–29). In our study, a particularly insightful finding is the significant correlation between severe endoscopic phenotypes, specifically ulceration, and distinct laboratory abnormalities, including elevated fibrinogen levels, increased platelet counts, and hypoalbuminemia. The association between endoscopic ulceration and elevated fibrinogen levels and platelet counts provides context for interpretation of potential pathophysiological mechanisms. However, our cohort did not include direct measurements of key complement mediators (e.g., C5a), and complement (C3/C4) and immunoglobulin profiles were available only in a subset of patients; therefore, mechanistic inferences should be interpreted cautiously. We propose that severe endoscopic phenotypes—particularly ulceration—may be compatible with immune pathway activation reported in prior studies, including complement involvement and neutrophil-mediated vascular injury. Elevated fibrinogen and platelet levels in these patients may reflect systemic inflammatory and coagulation responses accompanying more severe mucosal involvement, rather than directly demonstrating immunothrombosis in this cohort. Consistent with this, ulceration was associated with higher fibrinogen and platelet counts, whereas available immunoglobulin and complement levels did not differ significantly; however, because immune-marker testing was available only in subsets of patients, statistical power was limited and these results were interpreted cautiously without drawing mechanistic conclusions.

In renal sensitivity analyses, additional adjustment for renal involvement did not materially change the estimated associations between endoscopic features and LOS. This finding suggests that, within the constraints of the available laboratory markers, renal involvement did not explain the observed associations; however, renal disease severity may not be fully captured, and residual confounding cannot be excluded. Clinically, combining endoscopic findings with laboratory markers may provide a pragmatic multimodal assessment to support clinical evaluation, while acknowledging that treatment decisions and monitoring intensity may also influence LOS.

The clinical implications of these findings are substantial. The consistent associations observed between selected endoscopic features and LOS across models suggest these findings may be considered in early clinical assessment and monitoring, while avoiding interpretation as prognostic prediction. For instance, a patient presenting with acute abdominal pain and endoscopic evidence of extensive ulceration across multiple segments could be recognized as having features associated with longer hospitalization in this cohort, which may warrant closer observation in the appropriate clinical context. This early identification may support closer monitoring and earlier recognition of complications such as bleeding or perforation, and facilitate more efficient resource allocation. However, we caution that treatment escalation decisions were not standardized and may contribute to longer LOS. This approach is especially crucial for the subgroup of patients who present with prominent gastrointestinal symptoms prior to the classic emergence of purpura, a scenario often associated with diagnostic delays and potentially worse outcomes. Systematic endoscopic evaluation may help inform clinical decision-making by providing objective descriptors of GI involvement; however, its role should be viewed as supportive rather than prognostic given the observational design.

However, several methodological limitations of our study warrant careful consideration, as they contextualize the interpretation of our results and highlight directions for future research. The retrospective, single-center design inherently introduces the possibility of selection bias and unmeasured confounding, despite our efforts to adjust for key covariates statistically. First, our endoscopic assessment, while standardized and blinded, was based on a retrospective review by a single researcher, which may introduce classification bias compared to prospective, multi-observer consensus. Second, and critically, the reliance on standard upper and lower endoscopy without routine small-bowel evaluation (e.g., via capsule endoscopy) likely results in significant underestimation of jejunal and ileal involvement. This almost certainly leads to misclassification of some patients regarding multi-segment disease and attenuates the observed strength of its association with outcomes, highlighting the need for future studies with complete GI tract mapping. Additionally, the absence of systematic histological confirmation for endoscopic lesions prevents a definitive correlation between endoscopic appearances and histopathological evidence of leukocytoclastic vasculitis. While the clinical context strongly supports IgAV as the etiology, other causes of mucosal lesions cannot be entirely excluded without pathological verification.

When interpreting the association between endoscopic severity and prolonged hospitalization, it is essential to acknowledge potential non-medical and medical confounders. Factors such as institutional discharge protocols, prevailing practice patterns, socioeconomic status, and variations in management strategies (e.g., analgesic use, nutritional support) may independently affect the length of stay. Moreover, the potential influence of comorbid conditions and alternative diagnoses must be considered. Patients may have concurrent gastrointestinal pathologies such as inflammatory bowel disease, medication-related injury, or infectious enteritis that could independently prolong hospitalization. The presence of significant extra-intestinal manifestations, particularly severe renal involvement or arthritis, may also impact hospital stay independently of endoscopic findings. Although we excluded patients with known alternative diagnoses, subclinical comorbidities or early-stage concurrent conditions could potentially confound the observed relationships. These considerations reinforce the necessity of a comprehensive patient assessment when interpreting the prognostic implications of endoscopic findings.

Finally, our focus on hospitalization duration as the primary outcome, while clinically relevant for the acute disease phase, does not capture longer-term outcomes such as relapse rates, development of chronic sequelae, or quality of life. The prognostic value of these endoscopic features for long-term disease course remains an important area for future investigation with extended follow-up periods.

In conclusion, this study identifies endoscopic ulceration and multi-segment involvement (≥3 segments) as endoscopic phenotypes associated with longer hospitalization in patients with abdominal IgAV. These endoscopic phenotypes were also accompanied by laboratory features consistent with systemic inflammatory and procoagulant responses and were associated with a longer inpatient course. Given the observational design and the composite nature of LOS, these findings should be interpreted as associations rather than definitive predictions. We suggest that systematic endoscopic evaluation may be considered as part of the initial diagnostic workup and clinical assessment in patients with suspected IgAV presenting with gastrointestinal symptoms. The identified endoscopic features provide tangible descriptors that may support early clinical assessment; prospective validation is needed before any prognostic application. Future research should focus on the development and validation of a standardized endoscopic scoring system for IgAV in prospective, multicenter cohorts. This would facilitate consistent reporting, enhance comparability across studies, and ultimately contribute to optimizing individualized treatment strategies for this challenging patient population. Prospective, multicenter validation studies are warranted to confirm these findings and to develop a standardized endoscopic scoring system for clinical application, with the ultimate goal of improving outcomes in this severe disease phenotype.

## Data Availability

The raw data supporting the conclusions of this article will be made available by the authors, without undue reservation.
