# Supplementary material for: Endoscopic features associated with hospitalization outcomes in IgA vasculitis patients: a single-center retrospective cohort study
Source: Front Immunol. 2026 Apr 20;17:1731886. doi: 10.3389/fimmu.2026.1731886 (PMC13136241; doi:10.3389/fimmu.2026.1731886)
Supplement: Supplementary file 1 [file Table1.docx]

### Table S1. Comparison of laboratory indicators across Ulcerative vs. Non-ulcerative Lesions.

| **Items** | **Ulcerative Group** | **Non-ulcerative Group** | **P** |
| --- | --- | --- | --- |
| WBC | 15.38 ± 7.31 (n=35) | 13.25 ± 5.99 (n=96) | 0.128 |
| NEU | 13.10 ± 6.97 (n=35) | 10.86 ± 5.80 (n=96) | 0.070 |
| LYM | 1.44 ± 0.56 (n=35) | 1.56 ± 0.71 (n=96) | 0.478 |
| MONO | 0.75 ± 0.37 (n=35) | 0.72 ± 0.46 (n=96) | 0.337 |
| EOS | 0.08 ± 0.08 (n=35) | 0.09 ± 0.11 (n=96) | 0.464 |
| BASO | 0.03 ± 0.02 (n=35) | 0.03 ± 0.02 (n=96) | 0.725 |
| RBC | 4.87 ± 0.63 (n=35) | 4.79 ± 0.68 (n=96) | 0.303 |
| HGB | 143.46 ± 20.73 (n=35) | 142.80 ± 20.21 (n=96) | 0.660 |
| PLT | 345.17 ± 92.09 (n=35) | 306.07 ± 86.24 (n=96) | 0.026* |
| hsCRP | 26.65 ± 23.10 (n=5) | 18.05 ± 21.16 (n=19) | 0.783 |
| ESR | 75.00 ± 63.64 (n=2) | 13.92 ± 12.12 (n=12) | 0.082 |
| CRP | 31.33 ± 25.76 (n=7) | 49.18 ± 54.99 (n=5) | 0.465 |
| ALT | 19.38 ± 14.45 (n=34) | 18.89 ± 17.35 (n=97) | 0.975 |
| AST | 20.44 ± 11.42 (n=35) | 19.32 ± 7.83 (n=97) | 0.791 |
| ALB | 39.49 ± 5.61 (n=35) | 41.10 ± 5.96 (n=97) | 0.071 |
| GLO | 27.56 ± 5.75 (n=35) | 26.10 ± 4.46 (n=97) | 0.221 |
| TB | 12.06 ± 6.92 (n=35) | 12.55 ± 5.76 (n=97) | 0.391 |
| DB | 5.02 ± 3.22 (n=35) | 4.54 ± 2.44 (n=97) | 0.797 |
| GGT | 38.65 ± 35.91 (n=35) | 29.22 ± 34.17 (n=97) | 0.073 |
| ALP | 86.85 ± 35.36 (n=35) | 99.62 ± 61.52 (n=97) | 0.720 |
| Urea | 5.40 ± 3.68 (n=34) | 5.08 ± 2.21 (n=97) | 0.850 |
| UA | 312.59 ± 130.65 (n=35) | 330.16 ± 100.81 (n=97) | 0.268 |
| Crea | 72.19 ± 54.29 (n=35) | 59.75 ± 22.38 (n=96) | 0.269 |
| GFR | 120.03 ± 36.35 (n=35) | 134.57 ± 37.04 (n=97) | 0.116 |
| K | 4.20 ± 0.43 (n=35) | 4.21 ± 0.43 (n=97) | 0.951 |
| Na | 137.06 ± 3.78 (n=35) | 138.37 ± 3.46 (n=97) | 0.062 |
| Cl | 103.41 ± 4.29 (n=35) | 104.36 ± 5.93 (n=97) | 0.665 |
| Ca | 2.32 ± 0.17 (n=35) | 2.34 ± 0.18 (n=97) | 0.499 |
| PT | 13.34 ± 1.24 (n=35) | 12.99 ± 1.43 (n=96) | 0.192 |
| APTT | 32.86 ± 5.04 (n=35) | 31.13 ± 5.86 (n=96) | 0.074 |
| TT | 16.61 ± 1.32 (n=35) | 16.34 ± 1.47 (n=96) | 0.344 |
| D-Di | 6.72 ± 5.04 (n=14) | 5.75 ± 4.61 (n=37) | 0.624 |
| FDP | 27.23 ± 23.96 (n=14) | 22.43 ± 20.31 (n=37) | 0.458 |
| Fib | 4.79 ± 1.07 (n=35) | 3.92 ± 1.16 (n=96) | <0.001*** |
| IgA | 2.79 ± 0.84 (n=13) | 2.71 ± 1.02 (n=14) | 0.835 |
| IgG | 8.69 ± 2.58 (n=13) | 9.73 ± 2.22 (n=14) | 0.270 |
| IgM | 0.93 ± 0.59 (n=13) | 1.01 ± 0.51 (n=14) | 0.497 |
| IgE | 153.06 ± 213.44 (n=5) | 251.49 ± 246.31 (n=7) | 0.268 |
| C4 | 0.29 ± 0.12 (n=12) | 0.25 ± 0.10 (n=14) | 0.347 |
| C3 | 1.14 ± 0.23 (n=12) | 1.12 ± 0.26 (n=14) | 0.852 |
| ASO | 128.43 ± 79.64 (n=3) | 123.44 ± 119.84 (n=5) | 0.952 |
| RF | 9.56 ± 3.51 (n=5) | 6.58 ± 4.26 (n=4) | 0.080 |
| 24h UP | 2.34 ± 3.60 (n=7) | 0.80 ± 1.03 (n=19) | 0.184 |
| Urine Cr | 7765.70 ± 6124.65 (n=7) | 9926.89 ± 7343.91 (n=20) | 0.533 |
| Urine Alb | 1.01 ± 1.31 (n=7) | 0.58 ± 0.79 (n=20) | 0.361 |
| UPCR | 11.97 ± 29.64 (n=7) | 0.71 ± 0.86 (n=20) | 0.341 |
| Urine MA | 289.48 ± 415.22 (n=7) | 182.61 ± 318.86 (n=19) | 1.000 |
| UACR | 406.41 ± 534.58 (n=7) | 281.03 ± 512.44 (n=19) | 0.534 |

### Table S2. Comparison of laboratory indicators across Multi-segment (≥3 segments) vs. Limited-segment (≤2 segments) Involvement.

| **Items** | **≥3 segments** | **≤2 segments** | **P** |
| --- | --- | --- | --- |
| WBC | 15.50 ± 7.24 (n=38) | 13.06 ± 5.93 (n=92) | 0.058 |
| NEU | 13.27 ± 7.02 (n=38) | 10.63 ± 5.67 (n=92) | 0.035* |
| LYM | 1.35 ± 0.53 (n=38) | 1.61 ± 0.71 (n=92) | 0.066 |
| MONO | 0.77 ± 0.49 (n=38) | 0.71 ± 0.42 (n=92) | 0.572 |
| EOS | 0.09 ± 0.11 (n=38) | 0.09 ± 0.10 (n=92) | 0.973 |
| BASO | 0.03 ± 0.02 (n=38) | 0.03 ± 0.02 (n=92) | 0.700 |
| RBC | 4.59 ± 0.73 (n=38) | 4.90 ± 0.62 (n=92) | 0.012* |
| HGB | 137.92 ± 22.10 (n=38) | 145.13 ± 19.31 (n=92) | 0.057 |
| PLT | 337.61 ± 90.93 (n=38) | 306.07 ± 86.15 (n=92) | 0.064 |
| hsCRP | 19.04 ± 24.58 (n=6) | 20.11 ± 20.95 (n=18) | 0.581 |
| ESR | 12.75 ± 14.86 (n=4) | 26.60 ± 34.72 (n=10) | 0.322 |
| CRP | 38.69 ± 26.25 (n=4) | 38.81 ± 46.20 (n=8) | 0.808 |
| ALT | 21.78 ± 23.65 (n=38) | 17.63 ± 12.46 (n=92) | 0.328 |
| AST | 20.57 ± 10.00 (n=38) | 19.10 ± 8.38 (n=93) | 0.453 |
| ALB | 38.58 ± 5.41 (n=38) | 41.62 ± 5.83 (n=93) | 0.009** |
| GLO | 25.32 ± 3.57 (n=38) | 27.03 ± 5.22 (n=93) | 0.098 |
| TB | 11.24 ± 5.73 (n=38) | 12.99 ± 6.13 (n=93) | 0.071 |
| DB | 4.56 ± 2.87 (n=38) | 4.75 ± 2.59 (n=93) | 0.267 |
| GGT | 34.84 ± 38.34 (n=38) | 30.01 ± 33.22 (n=93) | 0.429 |
| ALP | 99.95 ± 57.29 (n=38) | 95.01 ± 55.87 (n=93) | 0.773 |
| Urea | 5.84 ± 3.98 (n=38) | 4.89 ± 1.84 (n=92) | 0.353 |
| UA | 314.19 ± 119.21 (n=38) | 332.24 ± 103.75 (n=93) | 0.173 |
| Crea | 70.64 ± 54.90 (n=38) | 60.15 ± 20.14 (n=92) | 0.949 |
| GFR | 125.57 ± 39.33 (n=38) | 132.79 ± 36.63 (n=93) | 0.711 |
| K | 4.18 ± 0.41 (n=38) | 4.21 ± 0.44 (n=93) | 0.731 |
| Na | 136.87 ± 2.91 (n=38) | 138.55 ± 3.71 (n=93) | 0.014* |
| Cl | 103.05 ± 4.33 (n=38) | 104.61 ± 5.92 (n=93) | 0.211 |
| Ca | 2.28 ± 0.17 (n=38) | 2.36 ± 0.17 (n=93) | 0.025* |
| PT | 13.24 ± 1.20 (n=38) | 12.99 ± 1.44 (n=92) | 0.351 |
| APTT | 32.04 ± 5.89 (n=38) | 31.41 ± 5.66 (n=92) | 0.826 |
| TT | 16.38 ± 1.29 (n=38) | 16.45 ± 1.48 (n=92) | 0.785 |
| D-Di | 7.55 ± 4.87 (n=14) | 5.40 ± 4.63 (n=36) | 0.194 |
| FDP | 30.06 ± 21.08 (n=14) | 21.29 ± 21.38 (n=36) | 0.159 |
| Fib | 4.26 ± 1.09 (n=38) | 4.12 ± 1.25 (n=92) | 0.554 |
| IgA | 2.44 ± 0.64 (n=6) | 2.84 ± 0.98 (n=21) | 0.369 |
| IgG | 7.44 ± 2.48 (n=6) | 9.75 ± 2.19 (n=21) | 0.036* |
| IgM | 0.72 ± 0.40 (n=6) | 1.04 ± 0.56 (n=21) | 0.201 |
| IgE | 62.75 ± 26.94 (n=2) | 240.02 ± 240.70 (n=10) | 0.273 |
| C4 | 0.30 ± 0.09 (n=6) | 0.25 ± 0.11 (n=20) | 0.294 |
| C3 | 1.09 ± 0.15 (n=6) | 1.14 ± 0.26 (n=20) | 0.641 |
| ASO | 155.00 ± 91.92 (n=2) | 115.42 ± 108.98 (n=6) | 0.664 |
| RF | 10.95 ± 0.49 (n=2) | 7.46 ± 4.17 (n=7) | 0.370 |
| 24h UP | 1.18 ± 1.41 (n=11) | 1.26 ± 2.62 (n=14) | 0.722 |
| Urine Cr | 8704.70 ± 4603.87 (n=9) | 9697.52 ± 8038.63 (n=18) | 0.940 |
| Urine Alb | 0.44 ± 0.58 (n=9) | 0.81 ± 1.08 (n=18) | 0.456 |
| UPCR | 0.59 ± 0.80 (n=9) | 5.15 ± 18.49 (n=18) | 0.527 |
| Urine MA | 241.50 ± 368.48 (n=8) | 198.00 ± 339.73 (n=18) | 0.216 |
| UACR | 404.95 ± 526.81 (n=8) | 274.71 ± 513.65 (n=18) | 0.216 |

### Table S3 Comparison of laboratory indicators across Active Bleeding vs. No Bleeding Signs.

| Item | Bleeding | No bleeding | P |
| --- | --- | --- | --- |
| WBC | 14.77 ± 7.36 (n=37) | 13.45 ± 6.00 (n=94) | 0.415 |
| NEU | 12.41 ± 6.95 (n=37) | 11.08 ± 5.86 (n=94) | 0.305 |
| LYM | 1.41 ± 0.66 (n=37) | 1.57 ± 0.67 (n=94) | 0.161 |
| MONO | 0.84 ± 0.60 (n=37) | 0.68 ± 0.35 (n=94) | 0.521 |
| EOS | 0.09 ± 0.10 (n=37) | 0.08 ± 0.11 (n=94) | 0.328 |
| BASO | 0.02 ± 0.01 (n=37) | 0.03 ± 0.02 (n=94) | 0.679 |
| RBC | 4.83 ± 0.80 (n=37) | 4.80 ± 0.61 (n=94) | 0.699 |
| HGB | 145.03 ± 23.48 (n=37) | 142.17 ± 18.94 (n=94) | 0.160 |
| PLT | 295.38 ± 100.89 (n=37) | 324.84 ± 83.25 (n=94) | 0.031* |
| hsCRP | 27.58 ± 26.41 (n=5) | 17.80 ± 20.16 (n=19) | 0.183 |
| ESR | 19.33 ± 12.74 (n=3) | 23.55 ± 34.20 (n=11) | 0.640 |
| CRP | 40.00 ± 64.22 (n=4) | 38.16 ± 25.70 (n=8) | 0.570 |
| ALT | 26.68 ± 24.39 (n=37) | 16.00 ± 11.05 (n=94) | 0.003** |
| AST | 23.37 ± 12.79 (n=37) | 18.16 ± 6.31 (n=95) | 0.035* |
| ALB | 38.90 ± 6.08 (n=37) | 41.36 ± 5.70 (n=95) | 0.039* |
| GLO | 25.65 ± 4.26 (n=37) | 26.82 ± 5.05 (n=95) | 0.250 |
| TB | 12.68 ± 5.94 (n=37) | 12.32 ± 6.14 (n=95) | 0.729 |
| DB | 5.21 ± 2.81 (n=37) | 4.46 ± 2.60 (n=95) | 0.231 |
| GGT | 42.64 ± 46.97 (n=37) | 27.46 ± 27.80 (n=95) | 0.006** |
| ALP | 86.32 ± 43.73 (n=37) | 100.09 ± 59.80 (n=95) | 0.247 |
| Urea | 5.04 ± 2.83 (n=37) | 5.21 ± 2.60 (n=94) | 0.389 |
| UA | 304.68 ± 98.50 (n=37) | 333.61 ± 112.63 (n=95) | 0.248 |
| Crea | 64.55 ± 25.43 (n=37) | 62.49 ± 37.15 (n=94) | 0.225 |
| GFR | 123.04 ± 28.41 (n=37) | 133.71 ± 39.95 (n=95) | 0.062 |
| K | 4.17 ± 0.47 (n=37) | 4.22 ± 0.41 (n=95) | 0.586 |
| Na | 137.76 ± 3.59 (n=37) | 138.13 ± 3.59 (n=95) | 0.602 |
| Cl | 104.97 ± 8.25 (n=37) | 103.77 ± 4.05 (n=95) | 0.496 |
| Ca | 2.27 ± 0.17 (n=37) | 2.36 ± 0.17 (n=95) | 0.006** |
| PT | 13.24 ± 1.29 (n=37) | 13.02 ± 1.42 (n=94) | 0.418 |
| APTT | 32.38 ± 6.20 (n=37) | 31.29 ± 5.48 (n=94) | 0.323 |
| TT | 16.25 ± 1.47 (n=37) | 16.48 ± 1.42 (n=94) | 0.400 |
| D-Di | 7.00 ± 5.36 (n=17) | 5.52 ± 4.34 (n=34) | 0.422 |
| FDP | 29.44 ± 25.43 (n=17) | 20.91 ± 18.56 (n=34) | 0.378 |
| Fib | 4.25 ± 1.16 (n=37) | 4.12 ± 1.22 (n=94) | 0.568 |
| IgA | 2.87 ± 0.77 (n=9) | 2.69 ± 1.01 (n=18) | 0.653 |
| IgG | 9.91 ± 1.68 (n=9) | 8.90 ± 2.68 (n=18) | 0.312 |
| IgM | 1.21 ± 0.63 (n=9) | 0.85 ± 0.47 (n=18) | 0.110 |
| IgE | 203.35 ± 221.06 (n=4) | 214.04 ± 247.16 (n=8) | 0.808 |
| C4 | 0.22 ± 0.11 (n=9) | 0.29 ± 0.10 (n=17) | 0.119 |
| C3 | 1.12 ± 0.26 (n=9) | 1.13 ± 0.24 (n=17) | 0.949 |
| ASO | 75.40 ± 6.55 (n=3) | 155.26 ± 120.56 (n=5) | 0.250 |
| RF | 7.10 ± 4.45 (n=4) | 9.14 ± 3.70 (n=5) | 0.802 |
| 24h UP | 1.17 ± 1.37 (n=9) | 1.25 ± 2.42 (n=17) | 0.914 |
| Urine Cr | 10325.88 ± 7157.82 (n=4) | 9199.75 ± 7122.27 (n=23) | 0.669 |
| Urine Alb | 1.48 ± 1.32 (n=4) | 0.55 ± 0.83 (n=23) | 0.070 |
| UPCR | 1.48 ± 1.32 (n=4) | 4.01 ± 16.40 (n=23) | 0.111 |
| Urine MA | 319.91 ± 396.84 (n=4) | 191.65 ± 337.70 (n=22) | 0.811 |
| UACR | 358.53 ± 412.28 (n=4) | 306.83 ± 534.65 (n=22) | 0.918 |

Abbreviations: WBC, White blood cell; NLR, Neutrophil-to-Lymphocyte Ratio; PLT, Platelet; PDW, Platelet Distribution Width; MPV, Mean Platelet Volume; CRP, C-Reactive Protein; FDP, fibrinogen degradation products;IgA: Immunoglobulin A; IgG: Immunoglobulin G; IgM: Immunoglobulin M; IgE: Immunoglobulin E; C3: Complement C3; C4: Complement C4; CRP: C-reactive protein; 24h UP:24-Hour Urine Protein; Urine β2-MG:Urine Beta-2-Microglobulin; Urine Cr:Urine Creatinine; Urine Alb:Urine Albumin; UPCR:Urine Protein-to-Creatinine Ratio; Urine MA:Urine Microalbumin; UACR:Urine Albumin-to-Creatinine Ratio.
